# The impact of irritable bowel syndrome on health-related quality of life in women with polycystic ovary syndrome

**DOI:** 10.1186/s12955-020-01428-7

**Published:** 2020-07-13

**Authors:** Fatemeh Bazarganipour, Seyed-Abdolvahab Taghavi, Zatollah Asemi, Helen Allan, Zahra Khashavi, Tahereh Safarzadeh, Shamsi Pourchangiz, Fatemeh Zare, Samaneh Ghasemi, Zivar Karimi, Maryam Azizi Kutenaee

**Affiliations:** 1 Social Determinants of Health Research Center, Yasuj University of Medical Sciences, Yasuj, Iran; 2 Medicinal Plants Research Center, Yasuj University of Medical Sciences, Yasuj, Iran; 3 Research Center for Biochemistry and Nutrition in Metabolic Diseases, Institute for Basic Sciences, Kashan University of Medical Sciences, Kashan, IR Iran; 4 Centre for Critical Research in Nursing & Midwifery, School of Health & Education, Middlesex University, London, UK; 5 Infertility Clinic, Omeleila Hospital, Bandar Abbas, Hormozgan Iran; 6 Student Research Committee, Hormozgan University of Medical Sciences, Bandar Abbas, Iran; 7 Fertility and Infertility Research Center, Hormozgan University of Medical Sciences, Bandar Abbas, Iran

**Keywords:** Polycystic ovary syndrome, Irritable bowel syndrome, Quality of life

## Abstract

**Background:**

The objectives of this study were to compare the prevalence and quality of life (QOL) of irritable bowel syndrome (IBS) in women with polycystic ovary syndrome (PCOS) compared with healthy women.

**Methods:**

This was a case-control study of 201 women recruited at an infertility clinic in Iran. The control group were healthy women (*n* = 100) and the comparison group, women with PCOS (*n* = 101). Data were collected by clinical Rome III criteria to determine the IBS, Bristol scale for stool consistency and IBS QOL.

**Results:**

The reporting of IBS symptoms were higher in PCOS (20.7%) than control group (11%) (*P* = 0.05). The IBS QOL score in the IBS + PCOS group was lower than other groups (IBS+ non PCOS, non IBS + PCOS, non IBS+ non PCOS; scores in food avoidance and worries about health domains were significant (*P* < 0.01).

**Conclusions:**

We conclude that having PCOS and an increased level of LH/FSH tends to cause IBS symptoms. IBS + PCOS women experience significant impaired quality of life scores particularly in relation to worries about health and food avoidance. These results offer further insights into IBS in PCOS women and their functional status and wellbeing.

## Introduction

Irritable bowel syndrome (IBS) is one of the most common disorders of the digestive system, with a 10–20% incidence globally [1]. People with IBS have sensitive intestines and suffer from intestinal muscle spasms in response to food, gas, and stress. These spasms can lead to abdominal cramps and pains, changes in bowel function, bloating, diarrhoea, and constipation [2]. Patients with IBS also experience higher rates of absence from work, disruption of interpersonal relations, including a reluctance to have sexual intercourse. IBS can prevent patients from attending social gatherings and travelling for fear of experiencing symptoms in public. Many studies have reported a lower level of the quality of life (QOL) for these patients than the general population [3–6].

Although studies have shown that the prevalence of IBS in women is twice that of men generally, it was believed that this disorder was lower in women with PCOS compared to healthy women. However a recent study reported an increased prevalence of IBS in women with PCOS compared to healthy women [7]. Polycystic ovarian syndrome (PCOS) is a common chronic endocrine disease which affects 5–10% of women of reproductive age. It is characterized by chronic anovulation, with clinical or laboratory signs of hyperandrogenism [8]. Obesity is prevalent in women with PCOS due to elevated androgen levels [9]. However, there is no detailed description of the relationship between IBS and women with PCOS symptoms in relation to raised androgen levels and raised body mass index (BMI) [7].

These factors influenced the decision to investigate the prevalence of IBS in women with PCOS compared to healthy women and to compare QOL in a group of women with PCOS and healthy women.

## Methods

### Design and data collection

This was a case-control study of women with PCOS who attended an infertility clinic at a hospital in Hormozgan Province, Iran from May to September 2014. This clinic is the only referral center for infertility in the province. The sample size (201) was calculated using Mathur, et al., (2010) information. Women with a confirmed diagnosis of PCOS (*n* = 300) were invited to participate in the study as a case comparison group. After explaining the study objectives and obtaining written consent from each woman, final participants were randomly allocated to the PCOS (*n* = 101); a control group of healthy women whose partners had a diagnosis of male infertility (*n* = 100) were approached in the same way. Inclusion criteria were include being 15–40 years of age [10–17]; married; absence of non-classic adrenal hyperplasia, thyroid dysfunction, hyperprolactinemia; non-smoking; absence of following warning signs that rejected IBS included extreme weight loss over the past few months, fever, nocturnal symptoms, severe chronic constipation, diarrhea, frequent vomiting, progressive dysphasia, a history of travel to areas of parasitic infections, family history of colon cancer, inflammatory bowel disease; not having cough (bronchitis) during the last 3 months; no problems in speaking or listening; Iranian; not taking any prescription medication (except allergy medications and occasional pain medications) for at least 3 months before entering the study; having two of the following Rotterdam diagnostic criteria:

1. Polycystic ovaries visualized on ultrasound scan (presence of 12 follicles or more in one or both ovaries and/or increased ovarian volume i.e., > 10 ml),
2. Clinical signs of hyperandrogenism (hirsutism score based on hirsutism score greater than 7 or obvious acne),
3. having an interval between menstrual periods > 35 days and/or amenorrhea, defined as the absence of vaginal bleeding for at least 6 months (i.e. 199 days).

The women were requested to complete the study measures in clinic.

### Measures

1. Menstrual history: all women were asked for the interval between menstrual periods during the preceding 12 months; results were categorized to < 21 days, 21–34 days, 35–60 days (oligomenorrhea), > 199 days (amenorrhea) and variable.
2. BMI: weight and height were calculated by weight/ height squared [kg/m2] in all women.
3. Body hair: Clinical assessment of hirsutism in all women was determined using the Ferriman-Gallwey Scoring System (F/G score). Nine body sites (the upper lip, chin, chest, upper back, lower back, upper abdomen, lower abdomen, arm, and thigh) were graded from 0 (no terminal hair) to 4 (severe hirsutism). Scores can range from zero to 36. A score of seven or above was considered positive for hirsutism [18].
4. Acne: the Global Acne Grading System (GAGS) was used to determine acne in all women. The GAGS assesses six locations on the face and chest/upper back, calculating surface area, distribution, and density of pilosebaceous units of lesions. Each of the six locations is graded separately on a scale 0-to-4, with the most severe lesion within that location determining the local score. The global score is a summation of all local scores [19].
5. Socio-demographic status: The study used years of formal education as a measure of socioeconomic status, categorized into five levels: no education, first level (1 to 5 years), second level (6–9 years), third level (10–12 years) and fourth level (more than 12 years). Donyavi et al., (2011) show that education is a good proxy measure for socioeconomic status for Iranians.
6. Laboratory measures: An overnight or 8 h fasting venous blood sample was obtained from each patient. Follicle-stimulating hormone (FSH) and Luteinizing hormone (LH) were assessed in all participants by ELIZA (DRG Instruments GmbH, Marburg, Germany).
7. ROME III diagnostic criteria: since IBS has no structural disorder or biochemical signs, the diagnosis is based on clinical signs. The ROME III criteria for the diagnosis of IBS (2006) includes intermittent abdominal pain on at least 3 days of the month in the last 3 months with at least two of the following three criteria combined: (i) abdominal pain or discomfort improves with defecation, (ii) abdominal pain or discomfort starts with a change in frequency of defecation, (iii) abdominal pain or discomfort start with a change in stool consistency (appearance). It should be noted that the above criteria must be present in the last 3 months before the diagnosis, as well as at least last 6 months from the onset of symptoms. The validity and reliability of these tools are approved in Iran [20].
8. IBS-QOL questionnaire was designed by Patrick, et al., (1998) and contains 34 items with a five degree Likert scale. There are eight domains: dysphoria, relationships, sexual concern, health worry, social reaction, body image, food avoidance, and interference with activity. Scores were between 0 to 100; a higher score in this tool represents a worse quality of life [21]. The validity and reliability of these tools are approved in Iran.
9. Bristol scale stool consistency: Bristol stool form scale is a medical aid designed to classify the form of human feces into seven categories including type 1: Separate hard lumps, like nuts (hard to pass); type 2: sausage-shaped, but lumpy; type 3: like a sausage but with cracks on its surface; type 4: like a sausage or snake, smooth and soft; type 5: soft blobs with clear cut edges (passed easily); type 6: fluffy pieces with ragged edges, a mushy stool; type 7: watery, no solid pieces, entirely liquid. Types one and two indicate constipation, with three and four being the ideal stools (especially the latter), as they are easy to defecate while not containing excess liquid, and five, six and seven tending towards diarrhea [22].

### Statistical analysis

Data were presented as mean ± Standard Deviation (SD) and frequency (percent) for quantitative and qualitative variables, respectively. Pearson Chi square test was used to compare demographic variables in the two groups. Ordinal demographic variables were compared between the two groups using the Mann-Whitney U test. Independent samples t-test was used to compare the means of in two groups.

### Ethical considerations

The Ethics Committee of the Hormozgan Medical University reviewed the study.

## Results

### The study sample

Over a period of 6 months, 201 women were recruited to the study. The socioeconomic and clinical characteristics of patients are presented in Table 1. There was a significant difference between the two groups in terms of PCOS related features (such as acne, hirsutism and menstrual pattern and LH/FSH ratio) (*P* < 0.001).

**Table 1:** Socio-demographic & clinical characteristics in patients

	PCOS (*n* = 101)	Control (*n* = 100)	*P* value
Age (year)	28 ± 4.92	29.68 ± 5.36	0.02
Education (year)	11.74 ± 3.53	10.77 ± 3.84	0.06
BMI	25.52 ± 4.70	24.53 ± 3.88	0.1
Occupation			
Occupied	18	19	0.82
Housewife	83	81	
Parity	0.16 ± 0.42	0.15 ± 0.35	0.74
Menstruation			
< 21	2 (1.98)	2 (2)	< 0.001
21–35	34 (33.66)	79 (79)	
35–60	17 (16.83)	6 (6)	
> 3 month	7 (6.93)	1 (1)	
Variable	41 (40.59)	12 (12)	
Hirsutism score	3.35 ± 3.15	0.58 ± 1.39	< 0.001
Acne score	4.20 ± 5.01	1.76 ± 3.8	< 0.001
LH/FSH	1.88 ± 1.52	0.93 ± 1.21	< 0.001

### IBS subtype & stool frequency

IBS cases in women with PCOS was 29.7% and the control group 11% (*P* < 0.01). The highest number of PCOS women placed in subtype of IBS – Constipation (20.8). According to Bristol stool consistency scale, women were in 2 ^rd^ and 3rd rank (sausage-shaped, but lumpy; and like a sausage but with cracks on its surface, respectively) that matches with IBS-Constipation relatively (Table 2).

**Table 2:** IBS subtype and bristol scale stool consistency in patients

	PCOS (*n* = 101)	Control (*n* = 100)	*P* value
IBS subtype			
No IBS	71 (70.3)	89 (89)	0.002
IBS-C	21 (20.8)	5 (5)	
IBS-D	4 (4)	2 (2)	
IBS-M	5 (5)	4 (4)	
Bristol scale stool consistency			
0	71 (70.3)	89 (89)	< 0.001
1	4 (4)	2 (2)	
2	7 (6.9)	3 (3)	
3	10 (9.9)	3 (3)	
4	5 (5)	1 (1)	
5	2 (2)	1 (1)	
6	2 (2)	1 (1)	
7	–	–	

### Comparison of sum scores of IBSQOL

There was significant difference in QOL scores between two groups (*P* < 0.001) mainly in food avoidance and worries about health (Table 3). In other word, in PCOS patients the lowest scores of QOL were belong to IBS subgroup. Similarly, in control group the lowest scores of QOL were belong to IBS subgroup. Moreover, the IBS+ PCOS patients had lower QOL scores than IBS+ non PCOS patients.

**Table 3:** comparison the IBS-QOL scores in patients

	PCOS (*n* = 101)		Control (*n* = 100)		*P* value^b^
	IBS	No IBS	IBS	No IBS	
Overall	60.71 ± 13.30	99.46 ± 2.98	74.13 ± 20.92	98.40 ± 6.02	< 0.001
*P* value^a^	< 0.001		< 0.001		
Dysphoria	61.30 ± 13.94	99.64 ± 2.96	75.94 ± 2.77	99.39 ± 6.04	< 0.001
*P* value	< 0.001		< 0.001		
Interference with activity	60.83 ± 12.96	99.64 ± 2.96	74.02 ± 20.70	98.35 ± 6.16	< 0.001
*P* value	< 0.001		< 0.001		
Relationship	61.38 ± 12.58	99.64 ± 2.96	75 ± 23.27	98.04 ± 6	< 0.001
*P* value	< 0.001		< 0.001		
Sexual	61.66 ± 14.28	99.64 ± 2.96	76.13 ± 12.97	98.31 ± 6.30	< 0.001
*P* value	< 0.001		< 0.001		
Health worry	60.05 ± 13.79	99.64 ± 2.98	73.48 ± 21.02	98.59 ± 5.78	< 0.001
*P* value	< 0.001		< 0.001		
Food avoidance	59.72 ± 13.80	99.64 ± 2.96	70.45 ± 21.20	98.31 ± 6.30	< 0.001
*P* value	< 0.001		< 0.001		
Social reaction	61.85 ± 13.36	99.64 ± 2.96	75 ± 21.83	98.52 ± 5.80	< 0.001
*P* value	< 0.001		< 0.001		
Body image	61.66 ± 13.90	99.64 ± 2.96	73.29 ± 20.17	98.31 ± 6.30	< 0.001
*P* value	< 0.001		< 0.001		

^a^Between IBS and no IBS in PCOS / control groups, separately

^b^Between PCOS and control groups with IBS

## Discussion

In this study, IBS cases in patients with PCOS (29.7%) by using Rome III criteria is higher than the values reported in the general population. Mathur et al. (2010) have also reported the IBS prevalence 41.7% by criteria Rome I in women with PCOS. Several studies in Iran have also reported the prevalence of IBS in different populations. The prevalence of IBS in Iranian blood donors was 5.6% [23]. The prevalence of IBS in 18,180 people in five cities in Tehran province was estimated to be about 1% by employing Rome III criteria [24]. Several issues may contribute to such the wide range reported in these studies which use different diagnostic criteria for IBS. Rome III is less restrictive then Rome II which demands patients to report their symptoms over longer periods. Compared to Rome III, a higher number of patients self diagnose with IBS using Rome II. Therefore, it’s seemed that the prevalence estimated by Rome III is lower than Rome II and I [25]. proposes using Rome III criteria in studies. Moreover, the role of socioeconomic status and cultural differences should be taken into account. Ho et al., 1998 suggested that the IBS in the urban group more than rural probably because the urban group reported a significantly greater influence of stress than the rural group. It is believed that the prevalence of IBS is less in developing countries (like Iran) compared to western countries [26, 27].

Increased levels of ovarian hormones decrease gastrointestinal transit [28]. Therefore women with IBS report the symptoms related to constipation more than men, except at the time of menstruation when hormone levels have reduced [29]. In the present study, the majority of patients with PCOS (20.8%) have IBS dominant constipation (IBS-C). It seems the cause of this variant of IBS is due to the high levels of hormones in PCOS which interference with bowel function. However, if only the hyper-androgenic condition is involved in the increased risk of IBS, it might be expected that IBS cases in the PCOS group should be less than the control groups.

Stress can affect the gastrointestinal function, so that the start and the severity of the symptoms of IBS are related to acute and chronic stress. Therefore, patients with IBS have hyperactivity to stress (excessive response of limbic system to the stress) [30]. A history of adverse life events and stress cause changes in the hypothalamic–pituitary–adrenal axis response to stress and inappropriate signaling of Corticotropin-releasing hormone as the most important factor in the increased prevalence of IBS [31]. Previous studies have reported that women with PCOS have more anxiety and stress which may cause a higher prevalence of IBS in these patients [32]. In addition to PCOS, an increase in the amount of LH/FSH was also an important predictor variable of IBS. In women with PCOS, the LH/FSH ratio was higher and it seems that an increase the perceived stress by patients may lead to increased sensitivity of the hypothalamic–pituitary–adrenal axis and LH and consequently a higher prevalence of IBS in these patients. Use of gonadotropin releasing hormone analogue leuprolide acetate which dramatically decreases LH which would be improves of chronic abdominal pain in women [33]..

IBS has a strong effect on the quality of life in women are diagnosed with it as it imposes substantial social and economic costs due to the need for medical care and absenteeism at work. One study in Iran investigated the economic burden of IBS and showed the cost of IBS in Iran about 2.8 million dollars and this is of great significant for Iranian population [34].

There are no specific biomarkers to assess the condition of patients with IBS. Thus there needs to be increased attention to the non-pathological markers in evaluating the impact of IBS as a chronic disease on well being, daily functioning and QOL. According Frank et al., 2002) patients with IBS have poor QOL compared to the general population; the same authors emphasize that IBS has a greater negative impact on QOL than asthma, gastroesophageal reflux disease (GERD) or migraine headaches. In the present study, QOL in women with IBS in both groups was significantly lower than in women without IBS. In both groups, worries about health and food avoidance were the areas of QOL most affected by IBS. Sung Kim et al. (2010) reported that the most affected areas were dysphonia, worries about health and the food avoidance [35]. These findings suggests that in our patients like Korean female with IBS, IBS patients suffer more from anxiety about their disease than impairment of social activity or relationship by bowel symptom.

Studies on women with PCOS using an IBS specific QOL tools have not been found identified in the literature; our study is the first to use IBS specific QOL measures in women with PCOS compared to healthy women. Studies using SF36 [36] and WHOQOL [37] to measure QOL minimize the influence of gastrointestinal symptoms on general QOL. Specific QOL tools associated with IBS have been designed and validated and include specific areas associated with QOL most affected by IBS.

### Limitations

There are some limitations in our study. The study population is from a limited region of Iran and is not be representative of the general population either in Iran or other countries. Moreover, there are unmeasured covariates that may play an important role in the association between IBS and PCOS, for example, psychological history; that IBS was self-indentified may be another limitation.

## Conclusion

As the results of this study show, having PCOS and an increased level of LH/FSH might cause IBS. IBS + PCOS patients experience significant effects on QOL particularly in relation to worries about health and food avoidance. These data offer insights for clinicians into IBS in women with PCOS and their functional status and wellbeing.

## Data Availability

The primary data for this study is available from the authors (Fatemeh Bazarganipour) on direct request.

## Abbreviations

QOL: Quality of life
IBS: Irritable bowel syndrome
PCOS: Polycystic ovary syndrome
BMI: Body mass index
GAGS: Global Acne Grading System
FSH: Follicle-stimulating hormone
LH: Luteinizing hormone
